# Experiences of managerial accountability in Ethiopia’s primary healthcare system: a qualitative study

**DOI:** 10.1186/s12875-020-01332-5

**Published:** 2020-12-06

**Authors:** Netsanet Fetene, Akshar Patel, Tibebu Benyam, Assefa Ayde, Mayur M. Desai, Leslie Curry, Erika Linnander

**Affiliations:** 1 Yale Global Health Leadership Initiative, Addis Ababa, Ethiopia; 2 Yale School of Public Health, New Haven, CT USA; 3 Reform and Good Governance Directorate, Federal Ministry of Health, Addis Ababa, Ethiopia; 4 Yale Global Health Leadership Initiative and Yale School of Public Health, 60 College Street, PO Box 208034, New Haven, CT 06520-8034 USA

**Keywords:** Accountability, Primary care, Ethiopia, Health management

## Abstract

**Background:**

Despite calls for improved accountability in global health systems, and a set of clear and consistent theoretical accountability frameworks, empirical descriptions of how accountability is experienced and enacted in low- and middle- income country (LMIC) settings is limited. Therefore, we sought to characterize how managers at all levels of Ethiopia’s primary healthcare system experience accountability in their daily practice.

**Methods:**

We conducted in-depth key informant interviews with 41 key stakeholders across 4 regions (Amhara, Oromia, Southern Nations Nationalities and Peoples, and Tigray) in the context of the Primary Healthcare Transformation Initiative (PTI). Consistent with the principles of grounded theory, our team used the constant comparative method to identify emergent themes related to concrete areas that could be targeted to allow an overall culture of accountability to flourish.

**Results:**

Emergent themes were: development of a shared understanding of system-wide accountability, streamlining of managerial reporting lines, strengthening of medico-legal knowledge and systems, and development of mechanisms for bottom-up accountability.

**Conclusions:**

Findings may be valuable to policymakers seeking to create more effective national accountability frameworks; practitioners and development partners seeking to strengthen implementation of evidence-based accountability systems and practices; and researchers aiming to develop meaningful, practical measures of accountability in public health.

## Background

Sustainable Development Goal (SDG) 16 emphasizes the importance of developing accountable and transparent institutions as a prerequisite for the achievement of human potential [1]. Accountability in public health, commonly defined as the procedures and processes by which interrelated actors justify and take responsibility for their activities [2], promotes answerability between different levels of the health system (bureaucratic accountability) and between the health system and the community (external accountability) [3]. Bureaucratic accountability mechanisms may be further differentiated into top-down systems (where lower tiers of the health system report to higher tiers) and bottom-up systems (where higher tiers of the system are responsive to lower tiers). Global models of accountability in public health highlight five interrelated building blocks of accountability: transparency in communication, inclusiveness in decision-making and owning consequences, management of performance, stakeholder participation in achieving a common goal, and responsiveness to requests and grievances [4, 5]. These building blocks are not necessarily distinct; significant overlap exists, as robustness in one domain cannot be achieved without a similar investment and level of attention to the others [2, 6]. Accountability in health systems helps to reduce abuse, assure compliance with procedures and standards, and improve organizational learning and performance [7].

Despite calls for improved accountability in global health systems [8], and a set of clear and consistent theoretical frameworks to support development of accountability, empirical descriptions of how accountability is experienced and enacted in low- and middle- income country (LMIC) settings are limited [9]. Introduction of the WHO accountability framework in Ethiopia targeting polio eradication led to significant improvements in polio surveillance and eradication in as little as 1 year [10]. However, we are not aware of evaluation of application or evaluation of such an accountability framework in the context of broader primary care systems strengthening efforts. A number of health systems strengthening efforts have focused on the development of top-down performance measurement and management systems [11], although data quality issues hamper these efforts [12], and there is evidence that these systems, if poorly implemented, have the unintended consequence of constraining both managerial problem solving [3] and external accountability and responsiveness [4]. Promising mechanisms to promote external accountability in LMIC settings are emerging (including, for example, the use of community scorecards and health facility committees), but uneven implementation requires greater attention [13, 14] and practical tools to promote accountability are lacking.

Therefore, to support translation from theoretical frameworks of accountability into such tools in primary healthcare, we sought to characterize how primary healthcare managers in LMIC settings experience accountability in their daily practice, based on in-depth interviews with practicing managers at multiple levels of the primary healthcare delivery system. We hope that results will be valuable to policymakers seeking to create more effective national accountability frameworks; practitioners and development partners seeking to strengthen implementation of evidence-based accountability systems and practices; and researchers aiming to develop meaningful, practical measures of accountability in health.

### Setting

Ethiopia served as an ideal setting in which to study accountability. The government’s Health Sector Transformation Plan (HSTP) [15], highlights major gaps in performance management and accountability and outlines a plan to improve the efficiency and effectiveness of health system performance across all levels of the health system through an Accountability Development Program. The corresponding district-level transformation document also emphasizes accountability, with focus on the concepts of transparency, community decision-making, performance management, and better stakeholder engagement [16].

In Ethiopia, the public healthcare system is organized into a decentralized administrative structure that includes the Federal Ministry of the Health (FMOH), Regional Health Bureaus (RHBs), Zonal Health Departments (ZHDs), District (*Woreda*) Health Offices, and health facilities. In the rural context, the woreda health office is responsible for the delivery of primary care to a population of approximately 100,000 persons through a network of health centers (approximately 5 per woreda), health posts (approximately 5 per health center), and the health extension worker program (two workers per health post). Some districts also include a primary hospital. Each region is also served by a regional hub of the Pharmaceutical Funds and Supply Agency (PFSA), which is responsible for delivering supplies and equipment to the health facilities.

This study was conducted in the context of the Primary Healthcare Transformation Initiative (PTI), a 4.5-year program (October 2015 – May 2020) to support the woreda transformation agenda by creating a culture of performance management and accountability for primary healthcare [17]. At the time of the study, the program was working in 19 rural zones across 4 regions of Ethiopia (Amhara, Oromia, Southern Nations Nationalities and Peoples [SNNP], and Tigray), and activities included the development, testing, and national scale-up of accountability measures (key performance indicators, woreda management standards, and community score cards).

## Methods

### Design and sample

We conducted face-to-face key informant interviews in June and July of 2018 with stakeholders across Ethiopia’s primary healthcare system to explore perspectives on accountability and use of existing accountability mechanisms. We used purposive sampling based on organizational roles to identify key stakeholders in management roles from each tier of the primary healthcare system (facility, woreda, zone, region) and across a diversity of geographies (balanced representation across Amhara, Oromia, SNNP, and Tigray regions). At the national and regional levels, we interviewed officials most closely responsible for the performance of the primary healthcare system. We then randomly selected two PTI-supported zones from each region, one woreda within each of those zones, and finally one health center within each of those woredas. In each site, we interviewed the head of the administrative unit or facility. Table 1 provides the breakdown of number of interviews conducted at each level and in which region.

**Table 1:** Respondents by the administrative level and geographic region

	Amhara	Oromia	SNNPR	Tigray	Total
Federal Ministry of Health					3
Regional Health Bureau	2	2	2	2	8
Zonal Health Department	2	2	2	–	6
Woreda Health Office	2	2	2	2	8
Health Center	2	2	2	2	8
Primary Hospital	1	1	1	1	4
Pharmaceutical Fund and Supply Agency (PFSA)	1	1	1	1	4
Total	10	10	10	8	41

### Data collection and analysis

We used an open-ended interview guide to structure conversations with key informants (Appendix 1). Interviews were conducted in a private room at the participants’ workplace by two primary interviewers [NF, TB] with prior experience and training in qualitative interviewing who were paired with a PTI staff member working in the region in which data were being collected. The primary interviewer provided information on the purpose of the study and what would be required of participants. We used verbal consent processes consistent with the minimal risk of the study and with our commitment to foster rapport in the local languages. Following verbal informed consent, interviews were recorded on handheld recording devices. The secondary interviewer used an interview reflection sheet (Appendix 1) to note non-verbal behaviors and other cues in tone and communication, and to debrief with the primary interviewer. Interviews were conducted until theoretical saturation was reached, using interviewer debriefing and the constant comparative methods analysis (described below) to continue to enroll participants until new ideas did not emerge through subsequent [18]. The study team determined they had reached saturation after 41 interviews.

The primary interviewer asked questions in both Amharic and English languages, with the option of translation by the secondary interviewer into the participant’s preferred language, if necessary. Of the 41 interviews, 2 were conducted in English, 3 in Tigrigna, and 36 in Amharic. Interviews lasted approximately 30 min. Interviews were translated and transcribed to generate English-language transcripts for coding and analysis. The study protocol, including recruitment, consent and data management processes, was approved by the Yale Human Subjects Committee and deemed exempt from continuing review.

Because was little empirical literature to guide our investigation and were not seeking to confirm existing an theory we worked according to the principles of grounded theory [19], iteratively developing and applying a code structure using the constant comparative method [20, 21]. Five team members participated in primary coding of the interviews. Two coders independently read and coded each transcript, reconciled divergent coding through negotiated consensus as described below, and updated the code structure as new constructs emerged. The interview reflection sheets were cross-referenced during this process to promote contextualization and interpretation of the statements included in the transcript. The final code structure (Appendix 2) was then systematically reapplied to all transcripts using qualitative Atlas.ti 8 software (Berlin, Germany) to facilitate data management and analysis. The use of an interdisciplinary team in generating a code structure and coding the data created space for unique insights from differing perspectives and multiple interpretations of the data. Differences in interpretation were resolved through regular team meetings with attention to ensuring that divergent codes, which are useful in refining the existing theory and increasing reliability and validity, are highlighted [22]. Final code reports were generated and distributed to the analysis team for identification and consolidation of themes.

## Results

We identified 4 emergent themes related to concrete areas that could be targeted to promote overall culture of accountability (Table 2): development of a shared understanding of system-wide accountability, streamlining of managerial reporting lines, strengthening of medico-legal knowledge and systems, and development of mechanisms for bottom-up accountability.

**Table 2:** Emergent themes and key findings

Themes	Key Findings
**Development of a shared understanding of system-wide accountability**	• Degrees of understanding about the link between accountability and performance are varied • Focus is on accountability for individual performance as opposed to system-wide constructs of accountability • Preserve the connection between rights and accountability; consider constraints on the ability of the worker or the organization to perform well
**Streamlining of managerial accountability lines for healthcare managers**	• Multiple, unsynchronized lines of accountability for healthcare managers exist • Politically-driven appointments for technical roles at all levels, from the woreda to the Federal Ministry of Health, are common • Poor merit-based appointment system creates lack of incentives for high performance
**Strengthening of medico-legal knowledge and systems**	• Patients lack knowledge of their legal rights in the case of a medical error • Providers and institutions are unaware of what constitutes a medical mistake from a legal perspective • Medico-legal capacity at both the individual and institutional levels needs strengthening
**Development of mechanisms for bottom-up accountability**	• Overall lack of responsiveness from higher levels in the health system • Current systems for routine oversight are limited and are not designed to be responsive to complaints or other early signals • Community and lower-level stakeholders should regularly evaluate higher-level health system entities

### Development of a shared understanding of system-wide accountability

Despite the national policy emphasis on accountability for primary healthcare performance, participants from all levels described varying degrees of understanding about the link between accountability and performance, and expressed a need for guidance regarding their role in accountability practices. Differentiating among key concepts such as roles and responsibilities was described as a challenge across the system, indicating shared operational understanding of system-wide accountability is lacking:

“There is confusion. What's a role, responsibility, what's accountability, what's authority? There are so many things being used in social science - but there is no clarity - we need to work on this issue so that we, at the end of the day, have performance improvement. If responsibility and accountability do not relate to performance, it's nonsense. So we need to interrelate it with performance.” (PFSA Respondent)

Responses generally focused on accountability for individual performance (and consequences for poor individual performance), as opposed to system-wide constructs of accountability:

“I can’t say accountability practices have been strong as there were some gaps in holding people accountable for their low performance. For example, if maternal death occurred in one cluster, then there is no practice of going all the way to uncover the responsible person. The same is for child death - we don’t dig enough to identify the responsible body and hold them accountable.” (Health Center Respondent )

Participants reflected on the complex nature of accountability, with multiple actors influencing the performance of the system and the individual. They described how poor responsiveness from the supply chain agencies may result in stock-outs at the facility level, lower-level structures may have to await strategic initiatives if funding decisions are held up at higher levels, and delays in staff salaries from the regional level influences worker motivation at the front lines. In this context, they advocated for the need to preserve the connection between rights and accountability, noting that efforts to improve accountability should consider constraints on the ability of the worker or the organization to perform well.

“Workers’ right and responsibilities should go hand in hand. Previously, there were challenges in our woreda as duty payments were not coming in timely and we could not pay their duty for 3 to 6 months straight. During such scenarios, it’s very difficult to force them to continue working. We can’t hold them accountable until we fulfil their rights.” (Woreda Health Office respondent)

### Streamlining of managerial accountability lines for healthcare managers

Participants consistently mentioned there are multiple, unsynchronized lines of accountability for healthcare managers. Multiple lines of accountability for a manager working in the primary health system present challenges to role clarity and priority-setting. For example, woreda health office managers described that conflicting priorities from the zonal health department head (technical oversight) and woreda administrator (administrative and financial oversight) can lead to frustration.

“Some people could be accountable to other tasks that are not their primary responsibilities. For example, being woreda health office head, I could be held responsible [by woreda administrator] for other issues that have nothing to do with health. If you see the reality, I am doing something else at my assigned kebele [local community], but I was not comfortable about that but I have to do it as I’ve to answer to my bosses. I’d rather be evaluated and held accountable to the activities that are stated on my job description and if that’s the case, I think I’ll deliver more. Otherwise, the health performance will be negatively affected if health leaders are too engaged in other activities.” (Woreda Health Office respondent)

Similarly, multiple layers and unsynchronized health and political accountability lines are also felt by managers at higher level of the health system. A Zonal health department head reflected on the challenges of being accountable ‘in many directions’:

“If a sector is accountable to a party (political), to the administrator and to the public, the sector may fail to understand its mandates well. I suggest accountability needs to be in a single channel. Putting the public at the center of our concern, we need to channel accountability in the sector’s line of management. Currently, we are accountable in many directions.” (Zonal Health Department respondent)

Others described specific challenges associated with one of these lines of accountability: politically-driven appointments for technical roles at all levels, from woreda to the FMOH. This quote highlights tensions between the multiple sets of expectations, including both formal and informal accountability:

**“**As a political nominee, I am accountable to many. I am accountable to the public as I am sitting here to serve them. As a government institution, I am accountable to my immediate supervisor, the bureau head. As a political nominee, the regional administrator has appointed me for this position and I am also accountable there. There are formal and informal accountabilities. I am also accountable to the mission and responsibilities I am given in the programs where I am working with my colleagues.” (Regional Health Bureau respondent)

Some managers described the consequences of the absence of a merit-based appointment system, which creates a lack of incentives to achieve high performance. A Woreda office head cautioned that only a merit-based system will foster improvements:

“You may find an excellent health office head who transformed the woreda health and yet [got] fired from work on the following day. On the other hand, you may find a head of the woreda health office staying for long time in his place without any visible change in performance in the woreda. It has to be merit based. Otherwise, we cannot expect changes to come.” (Woreda Health Office respondent)

### Strengthening of medico-legal knowledge and systems

Respondents described the desire for strengthening of medico-legal capacity at both the individual and institutional levels. At the individual level, they advocated for teaching about legal and ethical accountability during pre-service clinical education so that providers throughout the system are aware of how medical mistakes are accounted for.

“We should increase the capacity of our professionals. If we are to fully implement accountability which also could include litigation issues, the professionals have to protect themselves lest they will be victim. Hence, every professional should receive capacity building trainings [on legal and ethical issues] from the day they graduate and enter the system. It may be difficult to give training on every practice that exists, but we can consider complete training on both technical and ethical issues during their formal trainings.” (Primary Hospital respondent)

At the institutional level, participants described that healthcare providers do not fear malpractice lawsuits because patients lack knowledge of their legal rights in the case of a medical error. In addition, in places where malpractice lawyers and legal codes for clinical errors do exist, providers and institutions are unaware of what constitutes a medical mistake from a legal perspective. Thus, legal enforcement is hampered from both perspectives.

“We need to have clear guidelines and systems. There is no legal framework to ask a provider that did medical errors. I am not sure if the medico-legal procedure is endorsed or not. Providers are not aware often to what extent they are accountable … there should be a legal professional at institution level to entertain legal issues happening in health, as part of the structure.” ( Woreda Health Office respondent)

### Development of mechanisms for bottom-up accountability

Participants emphasized the importance of systems that allow the community and lower-level stakeholders to regularly evaluate higher-level health system entities, reflecting that the current primary healthcare system bottom-up accountability is inadequate. Participants called for the decentralization of evaluation, making it a feature of every level within the health system.

“The principle is that individuals need to be evaluated from both directions, that is, both the supervisor and the subordinate have to evaluate an individual. The service providers also need to be evaluated by the community using the community score card, and at the same time the supervisor also needs to evaluate the staff. If the staff is evaluated only by the supervisor, accountability can hardly be ensured. We can be sure to have a true civil servant only when the community has a say or has an opportunity to evaluate the service provision. In my opinion, this is one of the failed systems.” (Regional Health Bureau respondent)

In addition to calls for a more formal bottom-up evaluation system, managers noted overall lack of responsiveness from higher levels in the system, as characterized by this example in which a primary hospital manager noted his difficulties in attempting to engage in the ideation and decision-making processes with higher-level managers in the ZHD and RHBs.

“Decisions may take as long as five years…let alone to be involved in decision-making process. Even our requests may not be responded to in a timely manner, and this is one of the problems in terms of good governance. We forward ideas, but utilization of our ideas is not expected. Once we identify issues, the addressing of them by higher levels [regional health bureau] is usually lacking.” (Primary Hospital respondent)

At the same time, higher-level respondents expressed frustration at their inability to detect and follow-up on events in a proactive, responsive way. They pointed to limitations in current systems for routine oversight, which focus on reporting on a set of health system performance measures, and are not designed to be responsive to complaints or other early signals of issues. Because of what participants described as “weak links” in reporting, they are not able to respond until the problem is elevated, fostering experiences of firefighting and reactivity.

“A health facility may be high-performing based on the report it submits using the standard format, but the way staff are being handled in a facility is not being reported; there are also other issues that are not being reported. Sometimes, we take corrective actions in the facilities after a staff appeals a complaint when coming here. We respond after the complaint arises by the community also; these days, social media also fans the problems. What we are following up on using the report is limited; in fact, we can’t follow every detail by the routine report. This may create a weak link in sensitive matters that are related with accountability. We need to work more on these weak links. Even from the reports, we focus on critical areas only.” (Regional Health Bureau respondent)

Specifically, several managers described how they learn about health facility concerns during public feedback meetings rather than from their own staff.

“Usually bad deeds are kept hidden. Whenever the technical service providers make errors, they don’t report them. We receive such errors through public feedback sessions. We get a lot of information during the public forums. We hear surprising remarks in the forums that we could have even never imagined.” (Woreda Health Office respondent)

## Discussion

Despite global and national efforts to improve accountability for primary healthcare performance, little is known about how accountability is experienced by managers within the system. Through systematic qualitative inquiry, we have identified 4 emergent themes related to concrete areas that could be targeted to promote an overall culture of accountability: Development of a shared understanding of system-wide accountability, streamlining managerial reporting lines, strengthening medico-legal knowledge and systems, and developing mechanisms for bottom-up accountability. These findings can inform efforts to translate theoretical frameworks and national policies into meaningful health systems strengthening efforts. Our findings extend the existing literature regarding the importance of cultivating a culture of accountability in healthcare systems for improving quality of healthcare [23] and the community member’s role in improving public health, including holding government facilities accountable for provision of services [24].

Practical implications of our findings are four-fold. First, the concept of system-wide (vs. individual) accountability needs to be consolidated into a shared understanding, supported by pragmatic tools for performance measurement and improvement. This may include the scale-up of the community score card and increased use of key performance indicators, including the tracking of management standards [17]. Second, health governance needs to recognize that accountability in the truest sense may not be achieved until workers’ rights are protected. Third, future programming should be sensitive to the multiple lines of accountability that can place conflicting demands on health system managers [23]. Being able to disaggregate these lines and co-creating consistent priorities across health and other sector stakeholders will likely improve the accountability process for managers. Fourth, throughout health systems and health education, both physicians and patients need to understand the legal implications of clinical mistakes. A robust medico-legal system will empower all stakeholders to improve their activities and how they engage in healthcare practice. Legal empowerment through awareness and access to representation will allow patients and managers to hold poor providers accountable and protect good providers.

Our findings should be interpreted in the context of two limitations. First, social desirability bias may have influenced the interviews due to a fear of reprisal given the political environment at the time of the study [23]. We used several techniques to mitigate bias, including using probes to elicit details that would be difficult to misrepresent, encouraging respondents to share both positive and negative experiences, and assuring confidentiality [22]. Second, we did not collect information or explore hypotheses related to the role of participants’ gender, age, or educational background on their experiences of accountability. Instead, consistent with literature on organizational governance and accountability in LMIC contexts [25], our sampling approach was designed to optimize diversity and balance of organizational characteristics (tier and geography). Of note, our qualitative study was geographically circumscribed to Ethiopia, with the aim of understanding a complex concept within the context in which it occurs. We were able to identify and provide robust descriptions of four emergent constructs, but further research is needed to determine whether they are salient in other country contexts [26], and to evaluate relationships between these constructs toward a unifying framework or guiding theory [19].

## Conclusions

This study provides a rich and systematic description of how managers across Ethiopia’s primary healthcare system experience accountability in their day-to-day work, highlighting common challenges, and revealing a desire for improvement. Findings may be valuable to policymakers seeking to create more effective national accountability frameworks; practitioners and development partners seeking to strengthen implementation of evidence-based accountability systems and practices; and researchers aiming to develop meaningful, practical measures of accountability in health.

## Data Availability

The deidentified data underpinning this study are available upon request to the corresponding author.

## Appendix 1

### Interview guide and reflection prompts

*We are interested in understanding your views of accountability and practice between different levels of the Ethiopian healthcare system. We would like your permission to record this interview. This lets me listen carefully to you rather than taking notes and it will accurately capture our conversation. All information will be strictly confidential and no identifying information about you or your organization will be included on the transcript. Digital files with audio-recorded information will be deleted as soon as the transcripts have been reviewed for accuracy. If at any point you would like me to turn off the recorder, please let me know. You are free to decline to participate, to end our interview at any time for any reason, or to choose to skip a question.*

*(turn on recorder) Based on this description, are you willing to participate?*

1. Please describe your background and role within the primary healthcare sector?

- How long in the role, How long in healthcare overall, Main responsibilities

2. What does accountability for primary healthcare mean to you?

- Do you think it is important? Why/why not?

3. Who do you feel accountable to?
4 Please explain how you feel the accountability is practiced?

- Probe on multiple accountability lines and challenges
- Lower in the hierarchy: reports, sharing of information, performance planning, and review
- Supervisors or those higher up in the hierarchy
  - How transparent in communication
  - Are you involved in decision-making?
  - Do they manage performance
  - Do they engage stakeholders
  - Do they respond to your requests?

5. Please describe tools and processes for accountability that are working well?

- Do you use management standards, KPIs?
- Are there managerial and community level feedback systems?
- Probe on challenges and lessons

6. Please describe tools and processes for accountability that are not working well?

- Probe on challenges and lessons

7. What recommendations do you have for improved accountability in the primary care system?

- What would help you do a better job related to accountability?
- General recommendations for improving managerial accountability in the PHC system

8. Is there anything else you would like to tell me about your thoughts on accountability?

#### Interview Reflection Prompts

1. Please give a short collection of core findings from the interview you had just conducted. These five to ten bullet points should describe the essence of the participant’s account and highlight what is distinctive about it.
2. Were there any surprising findings?
3. Do you think the participant was being forthcoming with his/her answers?

## Appendix 2

### Final codebook

1. Experience
2. Role and responsibilities
3. Accountability (concept, practice, knowledge, measurement)
  3.1. Conceptualizing accountability
  4.1 Accountability practices
  5.1 Knowledge (awareness) on accountability
4. Accountability line
  4.1. Top down accountability
  5.1 Bottom up accountability
  6.1 Interdependence in accountability
  7.1 Measuring accountability
  8.1 Political accountability
5. Accountability domains
  5.1. Transparency in communication
  6.1 Inclusiveness in decisions
  7.1 KPI use
  8.1 Management standards (EHCRIG, WMS) use
  9.1 CSC use
  10.1 Other accountability methods (Strategic and Annual planning and performance review, BSC, BPR etc.) use
  11.1 Accounting performer and non-performer
  12.1 Supervision
  13.1 Data quality concern
  14.1 Technology use
  15.1 Stakeholder engagement
  16.1 Community ownership
  17.1 Feedback process (provision, receiving etc)
  18.1 Grievance process
  19.1 Non-responsiveness/Responsiveness
  20.1 Owning responsibility
  21.1 Adherence to rules and regulations
  22.1 One-to five networks
  23.1 Success and recognition
6. Challenges related to accountability
  6.1. Challenges for ensuring accountability (Equipment, supplies, staffing, infrastructure etc.)
  7.1 System for accountability (process, practices, existing tools,)
  8.1 Culture on accountability
  9.1 Enforcement problem on accountability laws and regulations
  10.1 Incentivizing accountability
  11.1 Value given to accountability (commitment, emphasis given to accountability issues)
  12.1 Budget/financial issues
  13.1 Staff motivation and retention mechanisms
7. Key quote

## Abbreviations

HSTP: Health Sector Transformation Plan
FMOH: Federal Ministry of the Health
LMIC: Low- or middle- income country
PFSA: Pharmaceutical Fund and Supply Agency
PTI: Primary Healthcare Transformation Initiative
RHB: Regional Health Bureaus
SDG: Sustainable Development Goals
SNNP: Southern Nations, Nationalities, and Peoples
ZHD: Zonal Health Departments
